# Immune-Related Adverse Events Predict the Efficacy of Immune Checkpoint Inhibitors in Lung Cancer Patients: A Meta-Analysis

**DOI:** 10.3389/fonc.2021.631949

**Published:** 2021-03-01

**Authors:** Donghui Wang, Cen Chen, Yanli Gu, Wanjun Lu, Ping Zhan, Hongbing Liu, Tangfeng Lv, Yong Song, Fang Zhang

**Affiliations:** ^1^ Department of Respiratory and Critical Care Medicine, Jinling Hospital, Nanjing Medical University, Nanjing, China; ^2^ Department of Respiratory and Critical Care Medicine, Jinling Hospital, The First School of Clinical Medicine, Southern Medical University, Nanjing, China; ^3^ Department of Respiratory and Critical Care Medicine, Jinling Hospital, Medical School of Nanjing University, Nanjing, China

**Keywords:** immune-related adverse events (irAEs), efficacy, immune checkpoint inhibitors (ICIS), lung cancer, meta-analysis

## Abstract

**Background:**

Immune-related adverse events (irAEs) have been reported to be associated with the efficacy of immunotherapy. Herein, we conducted a meta-analysis to demonstrate that irAEs could predict the efficacy of immune checkpoint inhibitors (ICIs) in lung cancer patients.

**Methods:**

Literature on the correlation between irAEs and the efficacy of immunotherapy in lung cancer patients were searched to collect the data on objective response rate (ORR), overall survival (OS), or progression-free survival (PFS) of the patients. These data were incorporated into the meta-analysis.

**Results:**

A total of 34 records encompassing 8,115 patients were examined in this study. The irAEs occurrence was significantly associated with higher ORR {risk ratio (RR): 2.43, 95% confidence interval (CI) [2.06–2.88], *p* < 0.00001} and improved OS {hazard ratio (HR): 0.51, 95% CI [0.43–0.61], *p* < 0.00001}, and PFS (HR: 0.50, 95% CI [0.44–0.57], *p* < 0.00001) in lung cancer patients undergoing ICIs. Subgroup analysis revealed that OS was significantly longer in patients who developed dermatological (OS: HR: 0.53, 95%CI [0.42–0.65], *p* < 0.00001), endocrine (OS: HR: 0.55, 95%CI [0.45–0.67], *p* < 0.00001), and gastrointestinal irAEs (OS: HR: 0.58, 95%CI [0.42–0.80], *p* = 0.0009) than in those who did not. However, hepatobiliary, pulmonary, and high-grade (≥3) irAEs were not correlated with increased OS and PFS.

**Conclusion:**

The occurrence of irAEs in lung cancer patients, particularly dermatological, endocrine, and gastrointestinal irAEs, is a predictor of enhanced ICIs efficacy.

## Introduction

Immunotherapy has led to unprecedented improvements in the life expectancy of cancer patients, particularly those with lung cancer, by priming the immune system to fight against the tumor cells. ICIs target cytotoxic T-lymphocyte-associated protein 4 (CTLA-4) and programmed cell death-1 (PD-1)/programmed cell death ligand-1 (PD-L1), which are critical molecules that negatively regulate the T-cell activity and aid the tumor cells in evading immune surveillance. However, owing to the heightened immune response, the irAEs caused by ICIs are frequent and ineluctable. A meta-analysis showed that the probability of irAE incidence ranges from 54% to 76% and varies according to the types of ICIs (1). Any organ system could be involved, including the skin, endocrine glands, gastrointestinal tract, liver, pulmonary, and, less commonly, the cardiovascular and central nervous systems. Although the underlying mechanism has not been completely elucidated, increased T-cell activity, B-cell-mediated autoantibody production, and cross-reactive tumoral antigenicity have been suggested to be involved in the occurrence of irAEs (2).

The irAEs inevitably affect the treatment and prognosis of the patients. Once these events occur, clinicians adopt different management strategies according to the types and grades of irAEs. The National Comprehensive Cancer Network (NCCN), American Society of Clinical Oncology (ASCO), and European Society of Medical Oncology (ESMO) have issued guidelines for the management of irAEs and have provided comprehensive general treatment algorithms for the clinicians (3, 4). In terms of prognosis, discontinuing ICIs may affect the life expectancy of the patients. However, the clinicians have noticed that patients who developed irAEs were more likely to benefit from ICIs. Meanwhile, theoretically, both antitumor and anti-self-adverse effects could result when the immunity is enhanced. Thus, there seems to exist a correlation between the occurrence of irAEs and the efficacy of immunotherapy.

A systemic review of melanoma has been conducted, which revealed that irAEs could predict survival and response in patients treated with ICIs (5), however, comprehensive meta-analyses focusing on lung cancer have not yet been performed. Herein, our work aimed to elucidate whether the occurrence of irAEs could predict the efficacy of ICIs in patients with lung cancer. Furthermore, we performed a subgroup analysis to decipher the association of organ-specific and grade-specific irAEs with the clinical outcomes of the patients.

## Materials and Methods

### Search Strategy

PubMed, Embase, and Cochrane Library databases from inception to October 15, 2020 were searched to locate eligible studies reporting the association between the efficacy of ICIs and irAEs in lung cancer patients undergoing immunotherapy. The search strategy was “[immune-related (Title/Abstract)] AND [adverse events (Title/Abstract)] AND {[lung cancer (Title)] OR [SCLC (Title)]}.” The citations of relevant articles were also reviewed in case of omission. The language was restricted to English, and conference abstracts were also included.

### Inclusion and Exclusion Criteria

The studies that met the following criteria were included in the review:

1. Involving patients who were diagnosed with lung cancer.
2. Involving patients who were treated with CTLA-4, PD-1, or PD-L1 inhibitors.
3. Studies reporting the association between irAEs and ICIs efficacy.
4. Studies that provided data on ORR, OS, or PFS of the patients.
5. Studies that provided the HR of OS or PFS in patients who developed irAEs versus patients without irAEs and 95% CI.
6. Studies published in the English language.

The exclusion criteria of the studies to be included in the review were as follows:

1. Studies that provided OS and PFS, but not HR, or provided HR and P-value, but not 95% CI.
2. Duplicated data or overlapping study populations (the most recent report was included).
3. The adverse events were not caused by the use of ICIs.

### Data Collection and Quality Assessment

DW and CC independently extracted the data from the included studies. The following data were extracted: author, year of publication, trial design, landmark analysis, sample size, irAE type and grade, ORR of patients with and without irAEs, HRs, and 95% CIs for OS or PFS of patients with global, organ-specific, and grade-specific irAEs. Several studies provided both multivariate and univariate HRs, and we selected the former. If studies provided HRs with and without landmark analysis, we chose the former. If studies provided the HRs of global as well as organ-specific irAEs, we opted for the former. If studies provided the HRs of both grade-specific and all-grade irAEs, we selected the latter. The Newcastle-Ottawa scale (NOS) criteria were applied to assess the quality of the included studies. Discrepancies were resolved by discussions among DW, CC, and YG.

### Data Analyses

Review Manager (RevMan) Version 5.4 (Nordic Cochrane Center, Copenhagen, Denmark) was used to perform the statistical analyses. The log HRs of irAEs versus non-irAEs were obtained by calculating RevMan. If the HR of non-irAEs versus irAEs was provided instead of the opposite comparison, the HR and 95% CI of irAEs versus non-irAEs were calculated by determining the reciprocal of the original HR and 95% CI (6). Heterogeneity was assessed by applying the chi-square test and I^2^ statistic. A chi-square *p*<0.05 or an I^2^>50% was considered to indicate significant heterogeneity (7). The random-effects model was used in case of significant heterogeneity (8); if not, the fixed-effects model was used. Publication bias was evaluated using Begg’s test and Egger’s test. If there is a significant publication bias, we will further use the trim and fill method to evaluate.

## Results

### Records Selection and Characterization of the Identified Studies

**Figure 1:**
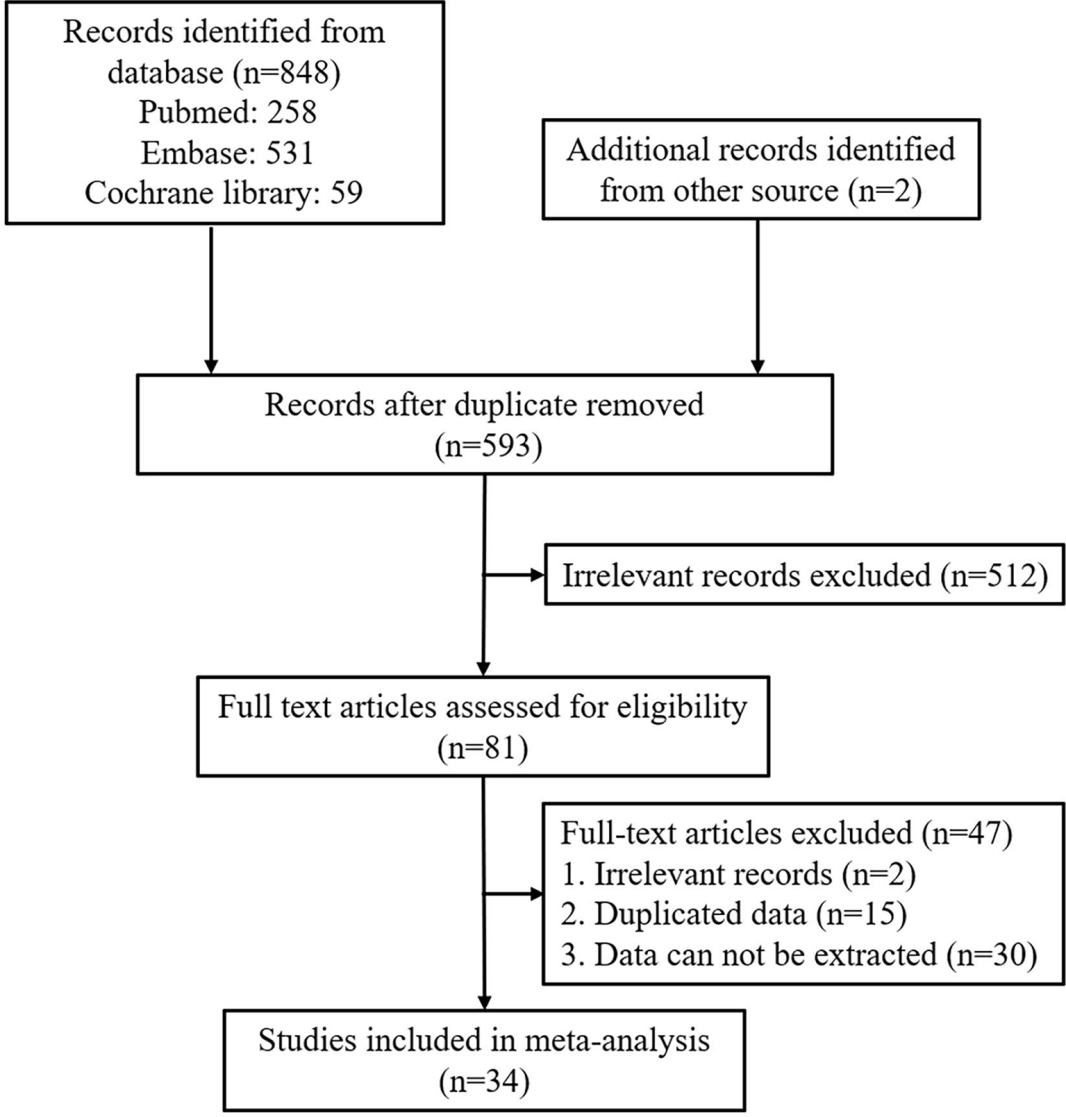
Flowchart of the study selection process.

A total of 848 records were retrieved from PubMed, Embase, and Cochrane Library databases, and two were acquired from additional resources. The flowchart of the records selection is given in **Figure 1**. After removing the duplicate records and excluding the irrelevant studies, 81 records were assessed for their eligibility. Finally, 34 records encompassing 8115 patients were chosen for this meta-analysis (9–42). Among the included studies, 31 had employed a retrospective cohort design, and 12 had adopted a landmark analysis. Twenty-one studies had reported the ORR of patients with or without irAEs, 23 had provided the HR of OS and 25 had provided the HR of PFS. Seventeen studies had reported organ-specific irAEs, and four had reported grade-specific irAEs. The characteristics of the included studies were listed in **Table 1** and **Supplementary Material 1 (Table S1)**.

**Table 1:** Main characteristics of the included studies.

Study	Design	ICIs types	Landmark analysis	irAEs types	irAEs grade	ORR (irAEs vs non-irAEs)	OS Hazard ratio (95%CI)	PFS Hazard ratio (95%CI)
Osorioet al. (9)	Retrospective	P	no	Thyroid dysfunction	1–3	NA	0.29 (0.09–0.94)	0.58 (0.27–1.21)
Teraoka et al. (10)	Prospective	N	6-week	Global	1–2	33.3%/12.5%	NA	NA
Kim et al. (11)	Retrospective	N/P	no	Thyroid dysfunction	1–2	31.6%/10.3%	0.11 (0.01–0.92)	0.38 (0.17–0.85)
Sato et al. (12)	Retrospective	N	no	Global	1–4	63.6%/7.4%	NA	0.10 (0.02–0.37)
Lisberg et al. (13)	Retrospective	P	no	Global	1–4	NA	0.75 (0.58–0.96)	0.75 (0.56–0.99)
Haratani et al. (14)	Retrospective	N	6-week	Global Skin Endocrine	1–4	52.3%/27.9%	0.285 (0.102–0.675) 0.209 (0.049–0.618) 0.504 (0.027–2.629)	0.542 (0.295–0.971) 0.476 (0.232–0.912) 0.237 (0.037–0.842)
Owen et al. (15)	Retrospective	NA	no	Global	1–5	NA	0.364 (0.203–0.649)*	NA
Grangeon et al. (16)	Retrospective	NA	no	Global Pneumonitis Thyroiditis Colitis Hepatitis	1–5	23.1%/5.7%	0.29 (0.18–0.46) 1.42 (0.45–4.54) 0.46 (0.25–0.86) 0.24 (0.03–1.73) 0.97 (0.3–3.08)	0.42 (0.32–0.57) 1.19 (0.52–2.70) 0.58 (0.39–0.85) 0.73 (0.35–1.50) 0.97 (0.45–2.08)
Ricciuti et al. (17)	Retrospective	N	no	Global Lung Gastrointestinal Endocrine Skin Hepatobiliary	1–4	43.5%/10%	0.38 (0.26–0.56) 0.46 (0.24–0.89) 0.5 (0.26–0.98) 0.45 (0.28–0.72) 0.8 (0.46–1.39) 0.94 (0.53–1.66)	0.48 (0.34–0.67) 0.56 (0.33–0.96) 0.52 (0.3–0.9) 0.59 (0.4–0.89) 0.57 (0.35–0.95) 0.72 (0.41–1.24)
Cortellini et al. (18)	Retrospective	NA	6-week	Global Endocrine Skin Gastrointestinal Hepatic	1–4	46.5%/25.7%	0.53 (0.41–0.69) 0.55 (0.37–0.83) 0.43 (0.27–0.70) 0.61 (0.38–0.98) 1.09 (0.48–2.45)	0.57 (0.45–0.72) 0.63 (0.45–0.89) 0.46 (0.31–0.69) 0.68 (0.47–1.01) 1.47 (0.72–2.96)
Toi et al. (19)	Retrospective	N/P	no	Global	1–4	51.5%/12.7%	0.42 (0.24–0.71)	0.45 (0.30–0.68)
Ahn et al. (20)	Retrospective	N/P	6-week	Global Skin Endocrine Pneumonitis	1–4	41.2%/26.7%	0.484 (0.255–0.919) 0.420 (0.162–1.087) 0.255 (0.051–1.288) 4.117 (1.420–11.942)	0.434 (0.256–0.735) 0.643 (0.350–1.180) 0.368 (0.132–1.028) 1.686 (0.618–4.597)
Berner et al. (21)	Prospective	N/P	no	Skin	NA	60%/14.6%	0.29 (0.12–0.71)	0.22 (0.09–0.49)
Fukihara et al. (22)	Retrospective	N/P	8-week	Pneumonitis	1–5	42.9%/25.4%	NA	NA
Baldini et al. (23)	Retrospective	N	no	Global	1–4	27.2%/16.5%	0.69 (0.58–0.82)*	0.69 (0.6–0.79)*
Cui et al. (24)	Retrospective	N/P/A/D	no	Pneumonitis	1–4	61.9%/29.9%	NA	NA
Naqash et al. (25)	Retrospective	N	no	Global Thyroid Pneumonitis Hepatitis Colitis/diarrhea Skin	NA	NA	0.65 (0.52–0.8) 0.79 (0.53–1.19) 1.35 (0.89–2.02) 1.18 (0.63–1.97) 0.65 (0.35–1.21) 0.67 (0.41–1.07)	0.69 (0.55–0.87) 0.98 (0.67–1.42) 1.36 (0.91–2.02) 0.75 (0.45–1.31) 0.65 (0.35–1.21) 0.57 (0.36–0.88)
Serrano et al. (26)	Retrospective	NA	no	Global	1–3	NA	0.53 (0.31–0.93)	0.44 (0.26–0.73)
Akamatsu et al. (27)	Retrospective	NA	no	Global	NA	48.4%/10.7%	0.45 (0.11–1.88)	0.30 (0.10–0.85)
Pawel et al. (28)	Retrospective	A	no	Global	1–4	NA	0.79 (0.60–1.05)	NA
Hosoya et al. (29)	Prospective	N	2-week	Global Rash Diarrhea	1–4	60.9%/12.8%	0.92 (0.47–1.79)	0.6 (0.36–0.99) 0.57 (0.33–0.99) 1.08 (0.46–2.5)
Boussageon et al. (30)	Retrospective	NA	12-week	Global	1–3	NA	NA	0.33 (0.14–0.76)
Sosa et al. (31)	Retrospective	NA	no	Global	NA	NA	0.18 (0.06–0.53)	NA
Rizzi et al. (32)	Retrospective	NA	6-week	Global	3–4	47.9%/30.0%	NA	NA
Aso et al. (33)	Retrospective	N/P	6-week	Skin	1–3	72.0%/24.0%	0.75 (0.4–1.43)	0.58 (0.33–0.99)
Riudavets et al. (34)	Retrospective	NA	2.4-month	Global Rash Endocrine	1–5	48.6%/23.2%	0.32 (0.22–0.46) 0.42 (0.23–0.75) 0.59 (0.34–1)	0.43 (0.28–0.64)
Kubo et al. (35)	Retrospective	NA	no	Global	NA	NA	NA	0.43 (0.24–0.76)
Lim et al. (36)	Retrospective	N	no	Global	1–4	31.6%/11.3%	0.5 (0.33–0.76)	NA
Noguchi et al. (37)	Retrospective	P	no	Global	1–4	NA	NA	0.33 (0.17–0.65)
Sugano et al. (38)	Retrospective	N/P/A	no	ILD	NA	62.5%/20.9%	NA	0.39 (0.19–0.77)
Usui et al. (40)	Retrospective	N	8-week	Skin rash	NA	22.2%/20.8%	NA	0.435 (0.105–1.196)
Ahmed et al. (39)	Retrospective	N/P	no	Thyroid	NA	NA	NA	0.38 (0.2–0.71)
Bjørnhart et al. (41)	Retrospective	N/P	no	Global	3–4	NA	0.47 (0.21–1.05)	0.71 (0.39–1.27)
Ksienski et al. (42)	Retrospective	N/P	6-week	Global	≥3	NA	2.29 (1.05–4.98)	NA

irAEs, immune-related adverse events; ORR, objective response rate; OS, overall survival; PFS, progression-free survival; CI, confidence interval; NA, not available; N, nivolumab; P, pembrolizumab; A, atezolizumab; D, durvalumab; ILD, interstitial lung disease.

*The HR and 95% CI was calculated through taking reciprocal.

### Association Between the Occurrence of irAEs and ORR, OS, and PFS

**Figure 2:**
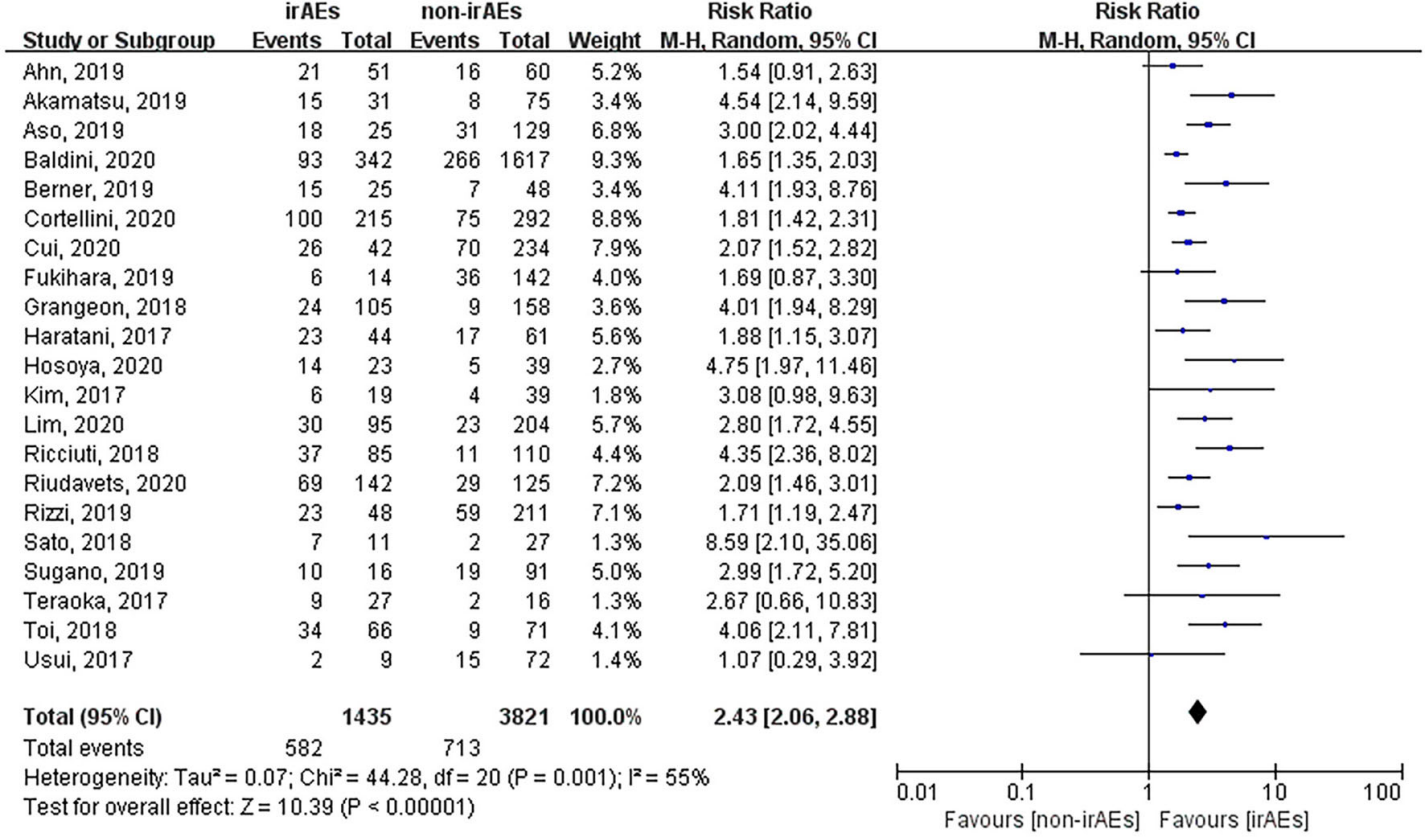
Forest plot of the association between the occurrence of irAEs and ORR. ORR, objective response rate; irAEs, immune-related adverse events; non-irAEs, non-immune-related adverse events.

**Figure 3:**
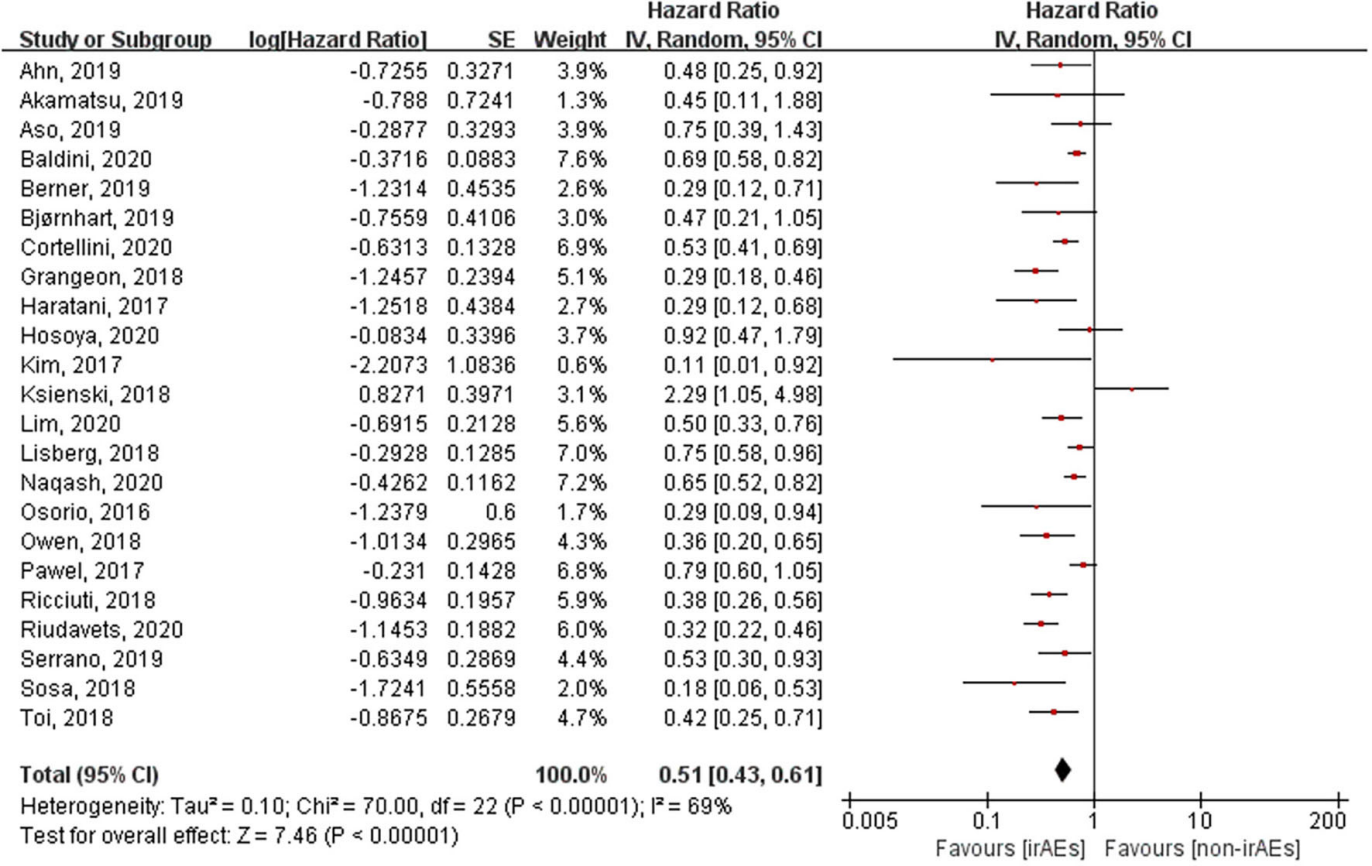
Forest plot of the association between the occurrence of irAEs and OS. OS, overall survival; irAEs, immune-related adverse events; non-irAEs, non-immune-related adverse events.

**Figure 4:**
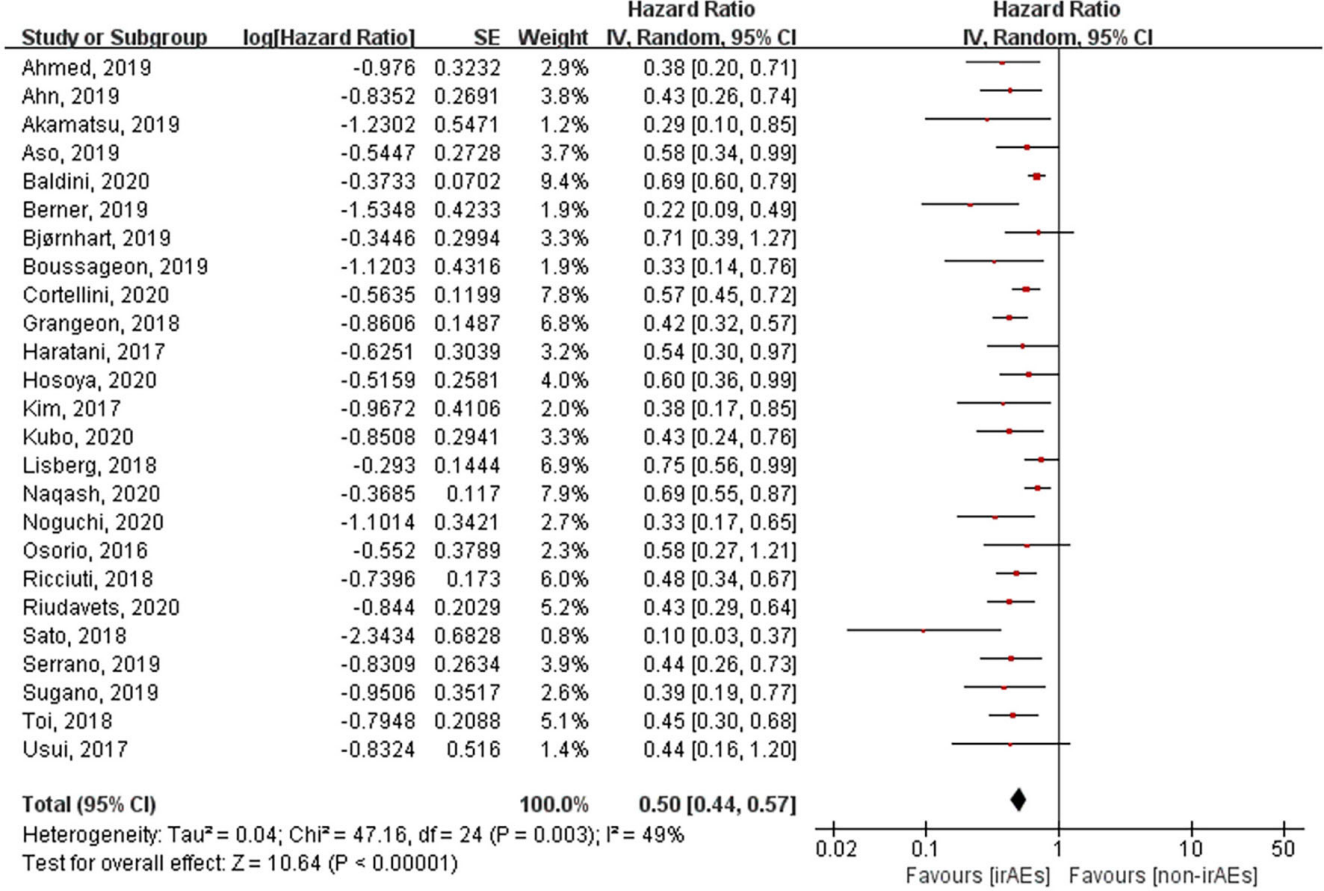
Forest plot of the association between the occurrence of irAEs and PFS. PFS, progression-free survival; irAEs, immune-related adverse events; non-irAEs, non-immune-related adverse events.

A total of 21 studies involving 5,256 patients had reported the ORR of patients with or without irAEs. The pooled RR for ORR was 2.43 (95% CI [2.06–2.88], *p* < 0.00001), which means that patients experiencing irAEs respond better to ICIs than those who do not (**Figure 2**). A random-effect model was utilized because of significant heterogeneity (I^2^ = 55%, *p* = 0.001). The occurrence of irAEs also predicted improved OS and PFS in lung cancer patients undergoing immunotherapy. The pooled HR OS was 0.51 for OS (95% CI [0.43–0.61], *p* < 0.00001) and 0.50 for PFS (95% CI [0.44–0.57], *p* < 0.00001). However, significant heterogeneities were observed for OS (I^2^ = 69%, *p* < 0.00001) and PFS (I^2^ = 49%, *p* = 0.003) (**Figures 3**, **4**).

### Subgroup Analysis

**Figure 5:**
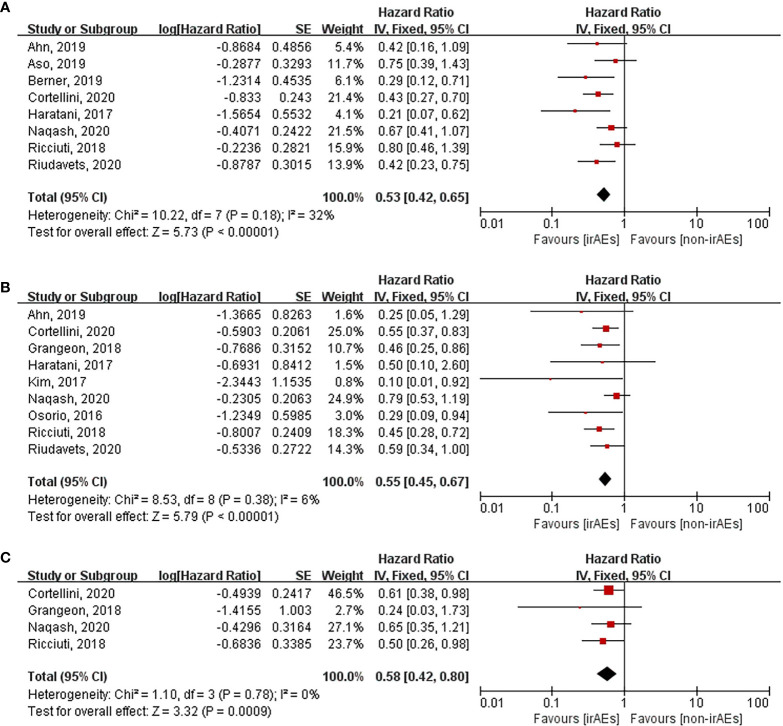
Forest plot of the association between the occurrences of different irAEs types and OS. **(A)** dermatological irAEs; **(B)** endocrine irAEs; **(C)** gastrointestinal irAEs. OS, overall survival; irAEs, immune-related adverse events; non-irAEs, non-immune-related adverse events.

**Figure 6:**
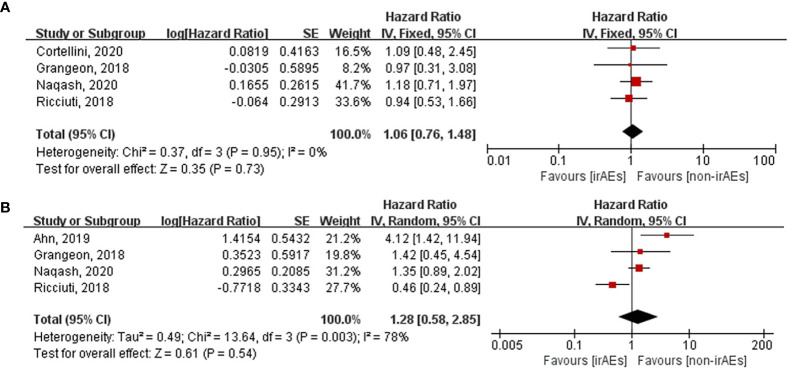
Forest plot of the association between the occurrences of different irAEs types and OS. **(A)** hepatobiliary irAEs; **(B)** pulmonary irAEs. OS, overall survival; irAEs, immune-related adverse events; non-irAEs, non-immune-related adverse events.

Various organ systems could be affected by irAEs. Zhou et al. had found that the association between the different irAE types and patient outcomes was inconsistent by conducting a meta-analysis that covered all cancer types (43). However, this issue remains unclear in case of lung cancer. Hence, we performed subgroup analysis according to irAE types and grades exclusively in lung cancer patients. After pooling the HRs of OS according to the irAE types, the occurrence of dermatological (HR: 0.53, 95% CI [0.42–0.65], *p* < 0.00001), endocrine (HR: 0.55, 95% CI [0.45–0.67], *p* < 0.00001), and gastrointestinal (HR: 0.58, 95% CI [0.42–0.80], *p* = 0.0009) irAEs was found to be significantly correlated with longer OS of lung cancer patients treated with ICIs (**Figure 5**). Nevertheless, no significant association was seen between OS and the occurrence of hepatobiliary (HR: 1.06, 95% CI [0.76–1.48], *p* = 0.73) and pulmonary (HR: 1.28, 95% CI [0.58–2.85], *p* = 0.54) irAEs (**Figure 6**). The association between irAE type and the PFS of patients was identical to that of OS (**Supplementary Material 1: Figures S1, S2**). Furthermore, we conducted a subgroup analysis based on the irAE grades. It was inferred that high irAE grades (≥ 3) were not significantly associated with OS and PFS (OS: HR:0.83, 95% CI [0.49–1.43], *p* = 0.51; PFS: HR: 0.88, 95% CI [0.67–1.16], *p* = 0.37) (**Supplementary Material 1: Figure S3**). Owing to data limitations, the association between low irAE grades (≤ 2) and patient outcomes could not be analyzed. In addition, we performed subgroup analysis based on the drug types. The pooled HR of OS was 0.57 (95%CI [0.45–0.72], *p* < 0.00001) for nivolumab monotherapy and 0.56 (95%CI [0.24-1.31], *p* = 0.18) for pembrolizumab monotherapy (**Supplementary Material 1: Figure S4**).

### Sensitivity Analysis and Publication Bias

Sensitivity analyses were conducted to assess the stability of our results. The results of OS and PFS remained consistent even upon deleting any included study or changing the random-effect model into the fixed-effect model (**Supplementary Material 1: Figures S5, S6**). The funnel plots assessing the publication bias of OS and PFS are presented in **Supplementary Material 1 (Figures S7, S8)**. Regarding OS analysis, Begg’s test (*p* = 0.369) revealed no publication bias, however, Egger’s test (*p* = 0.043) presented with the opposite result. We further adopted the trim and fill method to evaluate the publication bias. The result revealed that no study was trimmed or filled and that the pooled result of OS was unchanged, which indicated that our results were stable. Regarding PFS analysis, Begg’s test (*p* = 0.016) and Egger’s test (*p* < 0.001) suggested the existence of significant publication bias. The result of the trim and fill method was similar to that of OS.

## Discussion

Although the underlying pathophysiology has not been clearly elucidated until date, accumulating evidence suggest that the occurrence of irAEs can predict the outcomes of patients receiving immunotherapy. In our meta-analysis focusing on lung cancer, the results demonstrated that the occurrence of irAEs was significantly associated with ORR, OS, and PFS of patients treated with ICIs. In addition, patients who experienced dermatological, endocrine, and gastrointestinal irAEs had longer OS and PFS than those who did not. Nevertheless, pulmonary and hepatobiliary irAEs and high-grade (≥3) irAEs were not correlated with OS and PFS.

Several biomarkers have been investigated to predict the efficacy of ICIs prior to the treatment. Clinical practice suggests that not all predictors are effective. PD-L1 is the commonly adopted defective biomarker in clinical practice. Its expression in tumor cells, especially when ≥50%, was found to be significantly associated with the outcomes of lung cancer patients (44). Nevertheless, patients with negative PD-L1 expression also benefit from immunotherapy (45). Another acknowledged biomarker is the tumor mutational burden (TMB), whose predictive value is comparable to that of PD-L1 (46). Both tumor tissue and blood TMB have been shown to be associated with superior survival in lung cancer patients (47–50). However, technical limitations and the absence of standardization hampered its clinical application. Our results indicate that irAEs could be predictors of clinical outcomes in lung cancer patients after the initiation of treatment. These events are routinely witnessed within a few weeks of the initial administration, which helps clinicians in the early prediction of ICI efficacy. Most irAEs related to ipilimumab develop within 8–12 weeks of initiating the medication, with skin toxicity occurring at around 2–3 weeks, gastrointestinal and hepatic toxicity at 6–7 weeks, and endocrine toxicity at 9 weeks (51). The onset time of irAEs differs between anti-CTLA-4 and anti-PD-1 inhibitors. In patients treated with nivolumab, skin irAEs occur at about 5 weeks, followed by gastrointestinal irAEs at 7 weeks, hepatic irAEs at 8 weeks, pulmonary irAEs at 9 weeks, and endocrine irAEs at 10 weeks (51).

As mentioned previously, not all irAE occurrences are related to the efficacy of immunotherapy. The overall incidence of immune-related pneumonitis (IRP) was 4% (52, 53), with grade ≥3 accounting for 16.7%–42.9% of the cases (24, 25). A meta-analysis found that IRP was the major cause of death due to irAEs (54). Immune-related hepatitis (IRH) is usually asymptomatic, with elevated serum transaminase or bilirubin levels. Most patients developing IRH tended to receive corticosteroid treatment (55). For patients with grade ≥3 irAEs, which are usually life-threatening, ICIs should be permanently discontinued and treatment with immunosuppressive drugs such as glucocorticoids should be commenced. An increased risk of mortality and treatment with corticosteroids may together counteract the efficacy of ICIs.

While Zhou et al. had conducted a meta-analysis which mainly covered melanoma and non-small cell lung cancer to investigate the association between irAEs and the efficacy of ICIs (43), there are some key differences between our work and theirs. First, irAE profiles tend to vary according to the cancer type (56). For example, gastrointestinal irAEs occur more frequently in melanoma than in lung cancer, while the opposite is true for pneumonia. Therefore, the results obtained for other cancers may not be applicable for lung cancer. Although the cancer types studied by us are not as extensive as those investigated by Zhou et al., our results are more representative of the actual scenario in the specific field of lung cancer. Second, we pooled the RRs of ORR, which is lacking in Zhou et al.’s work. ORR is an important indicator of the response to immunotherapy, and our results demonstrated that patients experiencing irAEs respond better to ICIs than those who do not.

However, there are some limitations in our research. First, most of the included studies were retrospective, which might affect the quality of the evidences analyzed. Second, heterogeneities were significant in the analysis of ORR, OS, and PFS. After conducting a subgroup analysis based on the types of irAEs, the heterogeneities were significantly reduced. Third, Begg’s test and Egger’s test suggested the existence of significant publication bias. We further adopted the trim and fill method, which indicated that our results were stable. The last, but not the least, owing to the availability of only limited data, we were unable to analyze the association between low grade irAEs and patient outcomes. Hence, more cohort studies need to be performed in the future.

In conclusion, our meta-analysis has demonstrated that the occurrence of irAEs, especially dermatological, endocrine, and gastrointestinal irAEs, could predict the enhanced efficacy of immunotherapy in lung cancer patients treated with ICIs.

## Data Availability Statement

The original contributions presented in the study are included in the article/**Supplementary Material**. Further inquiries can be directed to the corresponding authors.

## Author Contributions

Conceptualization, supervision: FZ and YS. Data extraction: DW and CC. Writing – original draft preparation: DW. Methodology, software: YG and WL. Writing – reviewing and editing: PZ, HL, and TL. All authors contributed to the article and approved the submitted version.

## Funding

This work was supported by the National Natural Science Foundation of China (grant numbers 81970034 and 81570078).

## Conflict of Interest

The authors declare that the research was conducted in the absence of any commercial or financial relationships that could be construed as a potential conflict of interest.
