# Supplementary material for: Immune-Related Adverse Events Predict the Efficacy of Immune Checkpoint Inhibitors in Lung Cancer Patients: A Meta-Analysis
Source: Front Oncol. 2021 Mar 1;11:631949. doi: 10.3389/fonc.2021.631949 (PMC7958877; doi:10.3389/fonc.2021.631949)
Supplement: Supplementary file 1 [file DataSheet_1.docx]

**Supplementary Material 1**

Table S1: Main characteristics of the included studies.

| **Study** | **Tumor type** | **Patients’ number (irAEs vs non-irAEs)** | **irAEs types *** | **OS** | **PFS** |
| --- | --- | --- | --- | --- | --- |
| Osorio, 2016 | NSCLC | 10/38 | Thyroid dysfunction | Median: 40 vs 14 months | Median: 8 vs 2 months |
| Teraoka, 2017 | NSCLC | 27/16 | Global | NA | 4.1 [95% CI: 1.8–NR] VS 1.5 [95% CI: 1.2–2.3] months |
| Kim, 2017 | NSCLC | 19/39 | Thyroid dysfunction | NA | NA |
| Sato, 2018 | NSCLC | 11/27 | Global | NA | Median: NR [95% CI: 91 days to not applicable] VS 49 [95% CI: 36–127] days |
| Lisberg, 2018 | NSCLC | 39/58 | Global | NA | NA |
| Haratani, 2017 | NSCLC | 44/61 | Global | Median: NR [95% CI: 12.3 to NR] VS 11.1 [95% CI: 9.6 to NR] months | Median: 9.2 [95% CI: 4.4 to NR] VS 4.8 [95% CI: 3.0 to 7.5] months |
| Owen, 2018 | NSCLC | 27/64 | Global | Median: 24.3 [95% CI: 7.2 to NR] VS 5.3 [95% CI: 3.1-8.3] months | NA |
| Grangeon, 2018 | NSCLC | 105/158 | Global | Median: NR VS 8.21 [4.81-11.61] months | Median: 5.2 [3.7-6.88] VS 1.97 [1.79-2.16] months |
| Ricciuti, 2018 | NSCLC | 85/110 | Global | Median: 17.8 [95% CI: 11.6–24.1] VS 4.0 [95% CI 3.32–4.76] months | Median: 5.7 [95% CI: 4.18–7.38] VS 2.0 [95% CI 1.69–2.31] months |
| Cortellini, 2020 | NSCLC | 231/328 | Global | Median: NR VS 16.1 [95%CI: 13.6 – 27.4] months | Median:19.9 [95% CI: 16.4 – 26.3] VS 7.8 [95% CI: 6.5 – 9.8] months |
| Toi, 2018 | NSCLC | 66/71 | Global | Median: NR [95% CI: 14.5-NR] VS 11.4 [95%CI: 7.5-15.2] months | Median: 10.3 [95% CI: 5.5-15.2] VS 3.4 [95%CI:2.4-3.8] months |
| Ahn, 2019 | NSCLC | 96/59 | Global | 24.05 [95% CI: NR–NR) VS 7.39 [95% CI: 3.49–11.29] months | 11.63 [95% CI: 9.21–14.05] VS 3.27 [95%CI: 2.01–4.17] months |
| Berner, 2019 | NSCLC | 25/48 | Skin | NA | NA |
| Fukihara, 2019 | NSCLC | 27/124 | Pneumonitis | Median:3.4 VS 6.1 months | Median: 8.7 VS 23.0 months |
| Baldini, 2020 | NSCLC | 342/1617 | Global | Median: 16.7 [95% CI: 13.5–19.9] VS 9.0 [95% CI: 8.4–10.4] months | Median: 6.0 [95% CI: 4.9–7.1] VS 3.0 [95% CI: 2.8–3.1] months |
| Cui, 2020 | NSCLC | 42/234 | Pneumonitis | NA | Median: 45.08 [95%CI: 4.59-181.79] VS 21.15 [95%: 3.71-98.98] weeks |
| Naqash, 2020 | NSCLC | 173/531 | Global | Median: 14.9 VS 7.4 months | Median: 6.1 VS 3.1 months |
| Serrano, 2019 | NSCLC | 30/68 | Global | Median: 15.7 VS 6.1 months | Median: 7.7 VS 1.7 months |
| Akamatsu, 2019 | NSCLC | 31/75 | Global | 27.8 [95% CI: 10.5 to NR] VS 16.1 [95% CI: 6.8 to NR] months | 19.1 [95% CI: 5.5 to NR] VS 5.6 [95% CI: 1.6–9.9] months |
| Pawel, 2017 | NSCLC | 264/586 | Global | NA | NA |
| Hosoya, 2020 | NSCLC | 37/39 | Global | Median: 18.9 [95% CI: 8.6 to NR] VS 21.8 [95% CI: 8.1 to NR] months | Median: 5.0 [95% CI: 2.1-8.6] VS 2.0 [95% CI: 1.9-2.5] months |
| Boussageon, 2019 | NSCLC | 30/130 | Global | NA | NA |
| Sosa, 2018 | NSCLC | 15/49 | Global | NA | NA |
| Rizzi, 2019 | NSCLC | 48/211 | Global | Median: 29.4 [95%CI: NR] VS 12.9 [95%CI: 10.0 - 15.9] months | Median: 17.1 [95%CI: 8.1- 25.9] VS 6.6 [95%CI: 4.9 - 8.3] months |
| Aso, 2019 | NSCLC | 25/129 | Skin | Median: NR [95% CI: 17.5–NR]  VS 11.4 [95% CI: 8.8–15.6] months | median 12.9 (95% CI: 8.3 to not reached [NR]) VS 3.5 months (95% CI: 2.5–4.1) |
| Riudavets, 2020 | NSCLC | 142/125 | Global | 28.2 [95% CI: NR] VS 12.5 [95% CI: 10.8–14.2] months | 12.4 [95% CI: 1.9–22.9] VS 4.1 [95% CI: 2.6–5.6] months |
| Kubo, 2020 | NSCLC | 62/48 | Global | NA | NA |
| Lim, 2020 | NSCLC | 95/204 | Global | NA | NA |
| Noguchi, 2020 | NSCLC | 63/31 | Global | NA | Median: 371[95% CI: 184-NR] VS 67 [95% CI:51–87] days |
| Sugano, 2020 | NSCLC | 16/91 | ILD | NA | Median: 15.9 [95% CI: 5.0 to 18.8] VS 3.3 [95% CI: 2.1 to 5.9] months |
| Usui, 2017 | NSCLC | 9/72 | Skin rash | NA | Median: 8.0 VS 4.9 months |
| Ahmed, 2019 | NSCLC | 38/147 | Thyroid | NA | Median: 9.0 [95% CI: 9.3-17.1] VS 2.0 [95% CI, 2.0-13.5] months |
| Bjørnhart, 2019 | NSCLC | NA | Global | NA | NA |
| Ksienski, 2018 | NSCLC | 116/155 | Global | NA | NA |

Abbreviations: irAEs: immune-related adverse events; NSCLC: non-small cell lung cancer; OS: overall survival; PFS: progression-free survival; CI: confidence interval; NA: not available; NR: not reached; ILD: interstitial lung disease

* The irAEs types listed in this column are different from those in Table 1, mainly corresponding to OS and PFS in this Table.


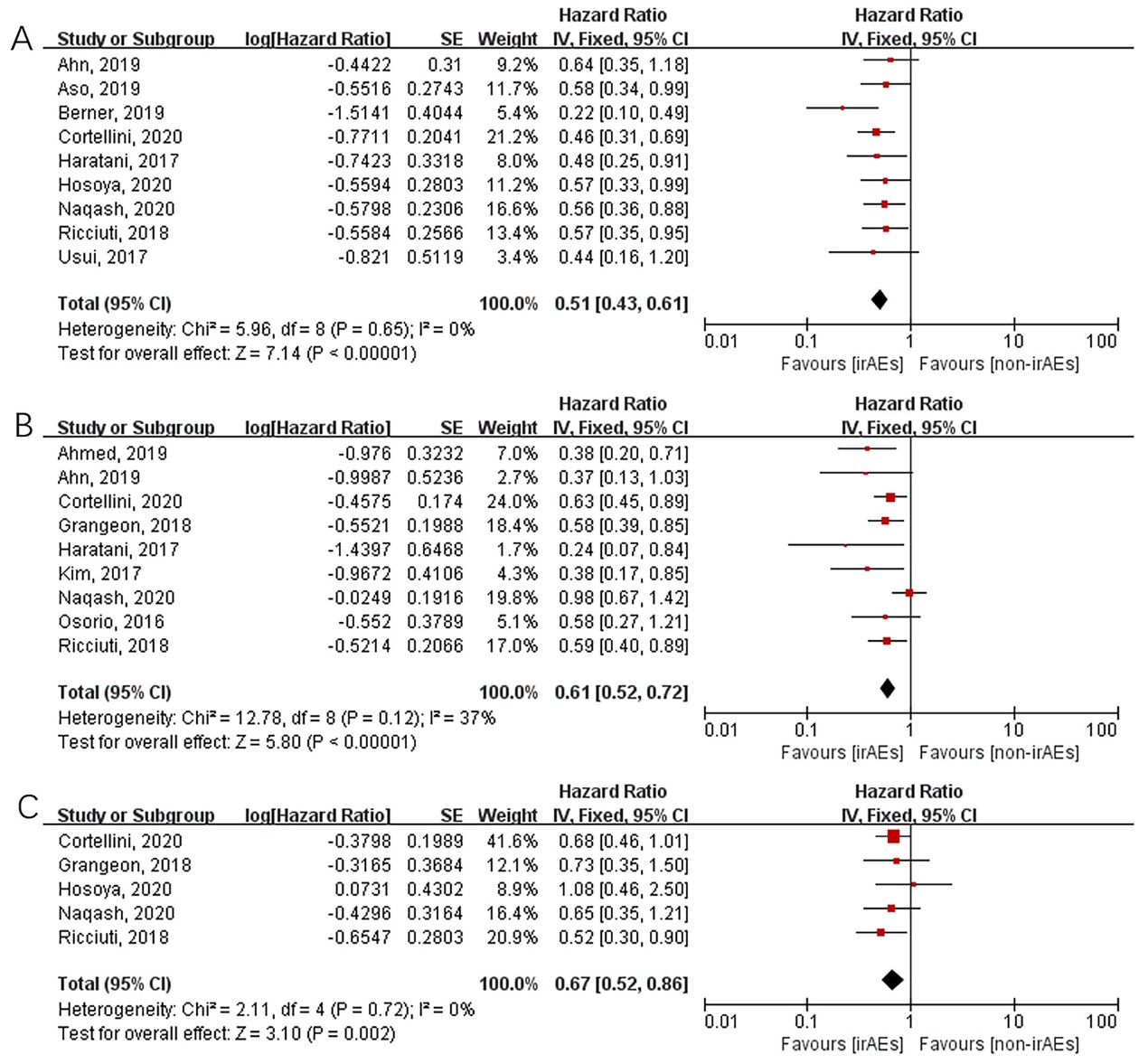


Figure S1: Forest plot of the association between the occurrences of different irAEs types and PFS. (A) dermatological irAEs; (B) endocrine irAEs; (C) gastrointestinal irAEs. Abbreviations: PFS, progression-free survival; irAEs, immune-related adverse events; non-irAEs, non-immune-related adverse events.


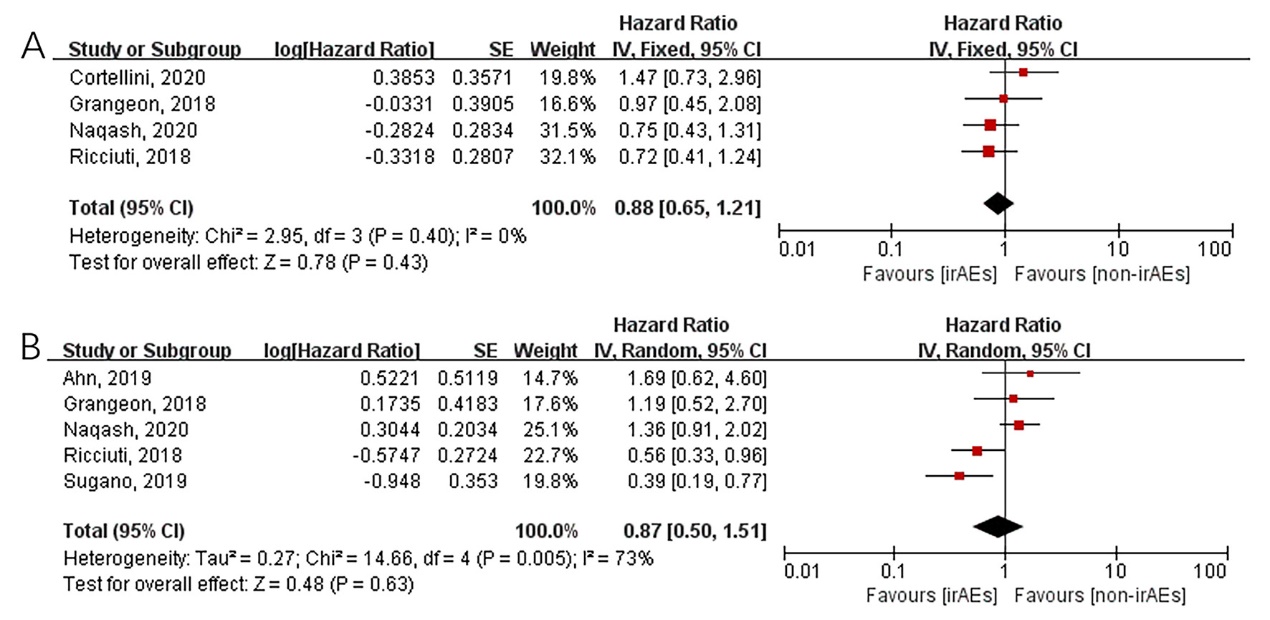


Figure S2: Forest plot of the association between the occurrences of different irAEs types and PFS. (A) hepatobiliary irAEs; (B) pulmonary irAEs. Abbreviations: PFS, progression-free survival; irAEs, immune-related adverse events; non-irAEs, non-immune-related adverse events.


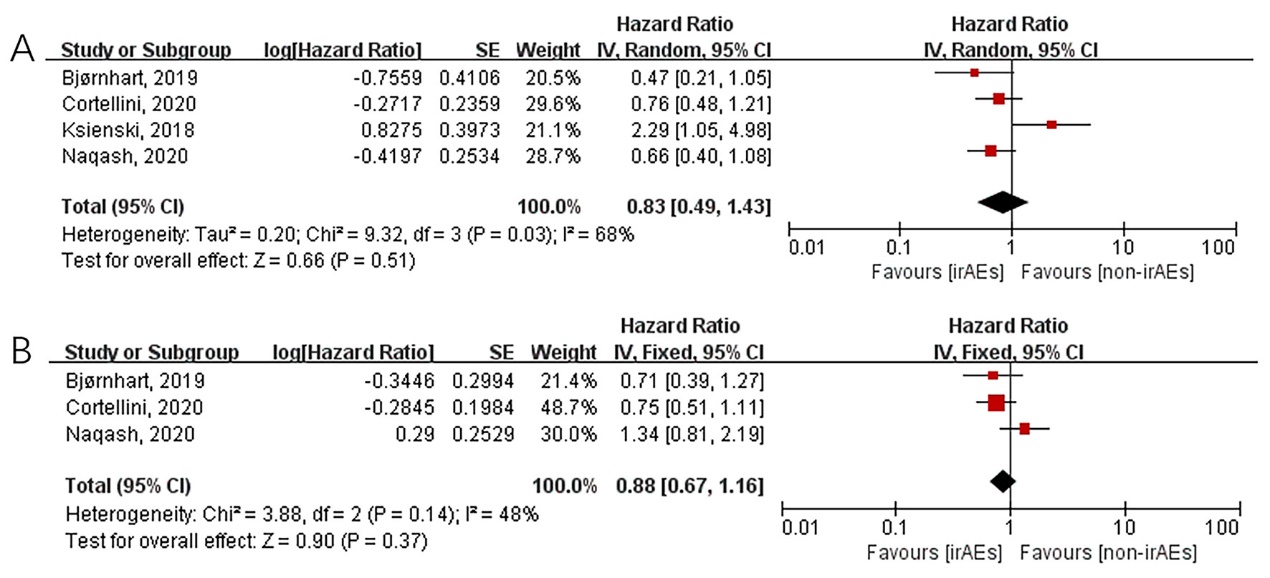


Figure S3: Forest plot of the association between the occurrences of high grades (≥3) irAEs types and OS and PFS. (A) OS; (B) PFS. Abbreviations: OS, overall survival; PFS, progression-free survival; irAEs, immune-related adverse events; non-irAEs, non-immune-related adverse events.


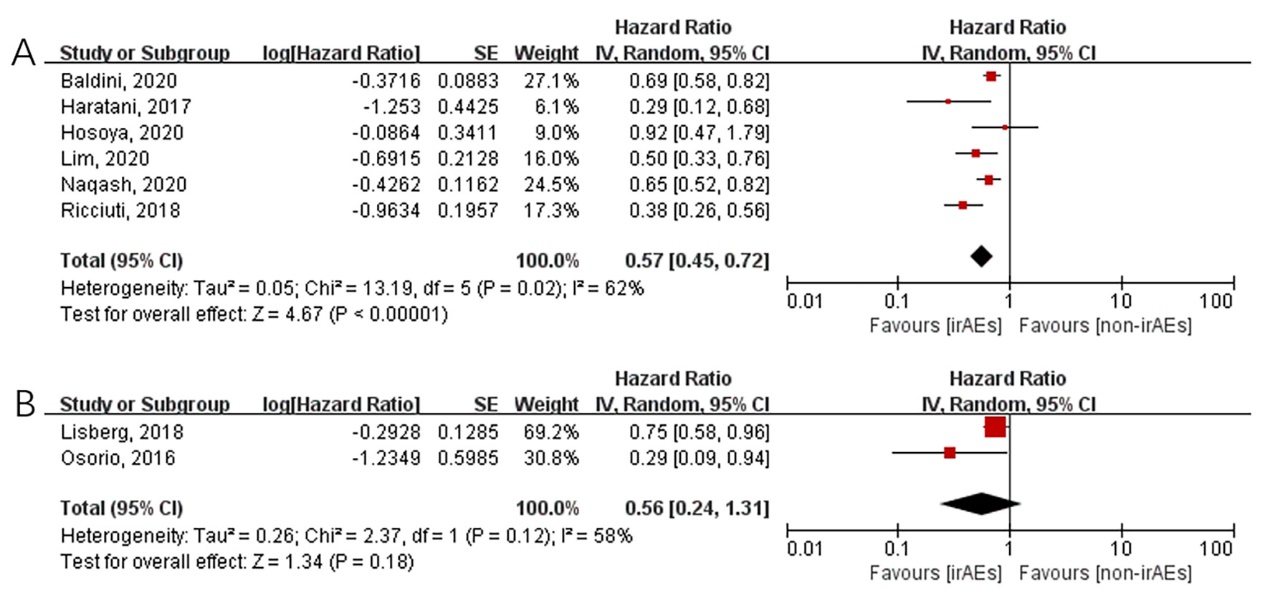


Figure S4: Forest plot of the association between the occurrences of irAEs and OS in patients receiving nivolumab and pembrolizumab. (A) nivolumab; (B) pembrolizumab. Abbreviations: OS, overall survival; irAEs, immune-related adverse events; non-irAEs, non-immune-related adverse events.


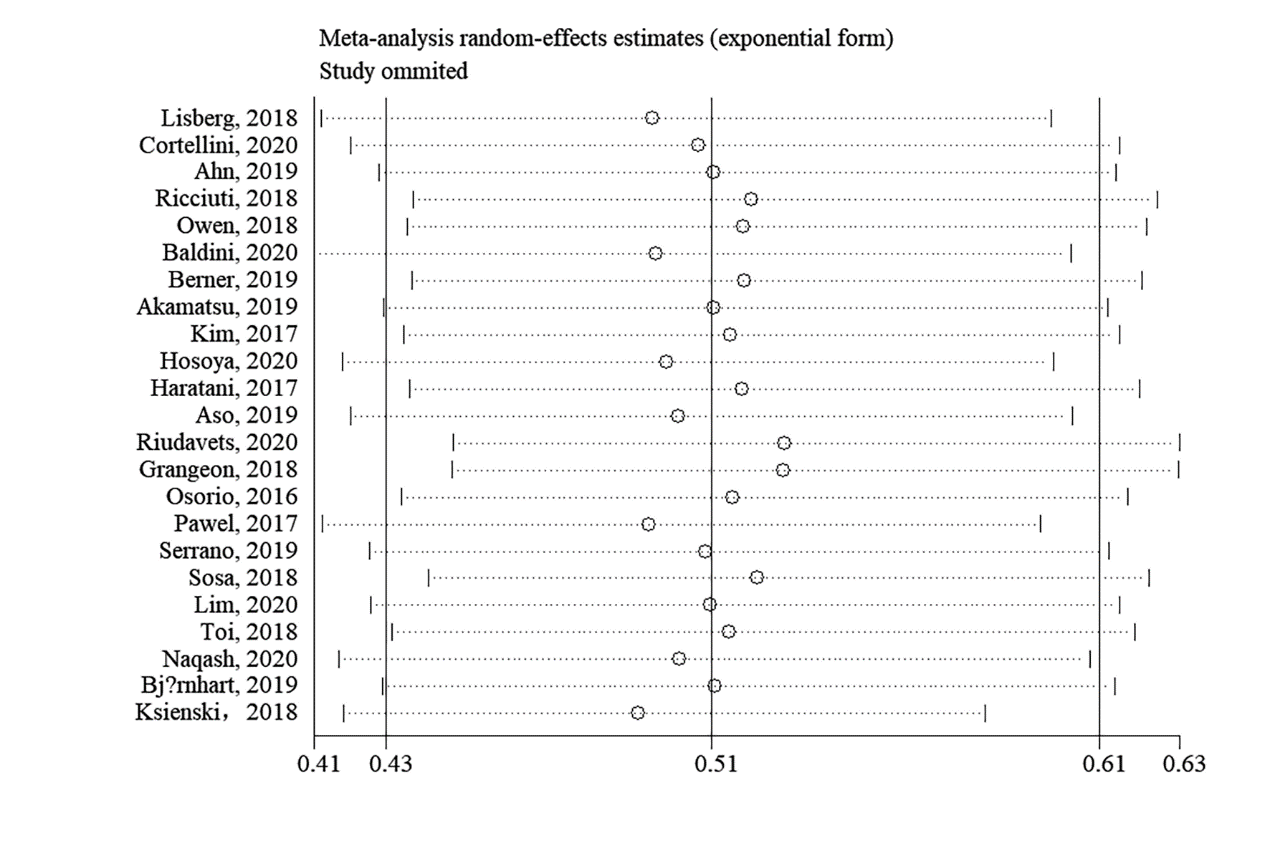


Figure S5: Sensitivity analysis of overall survival.


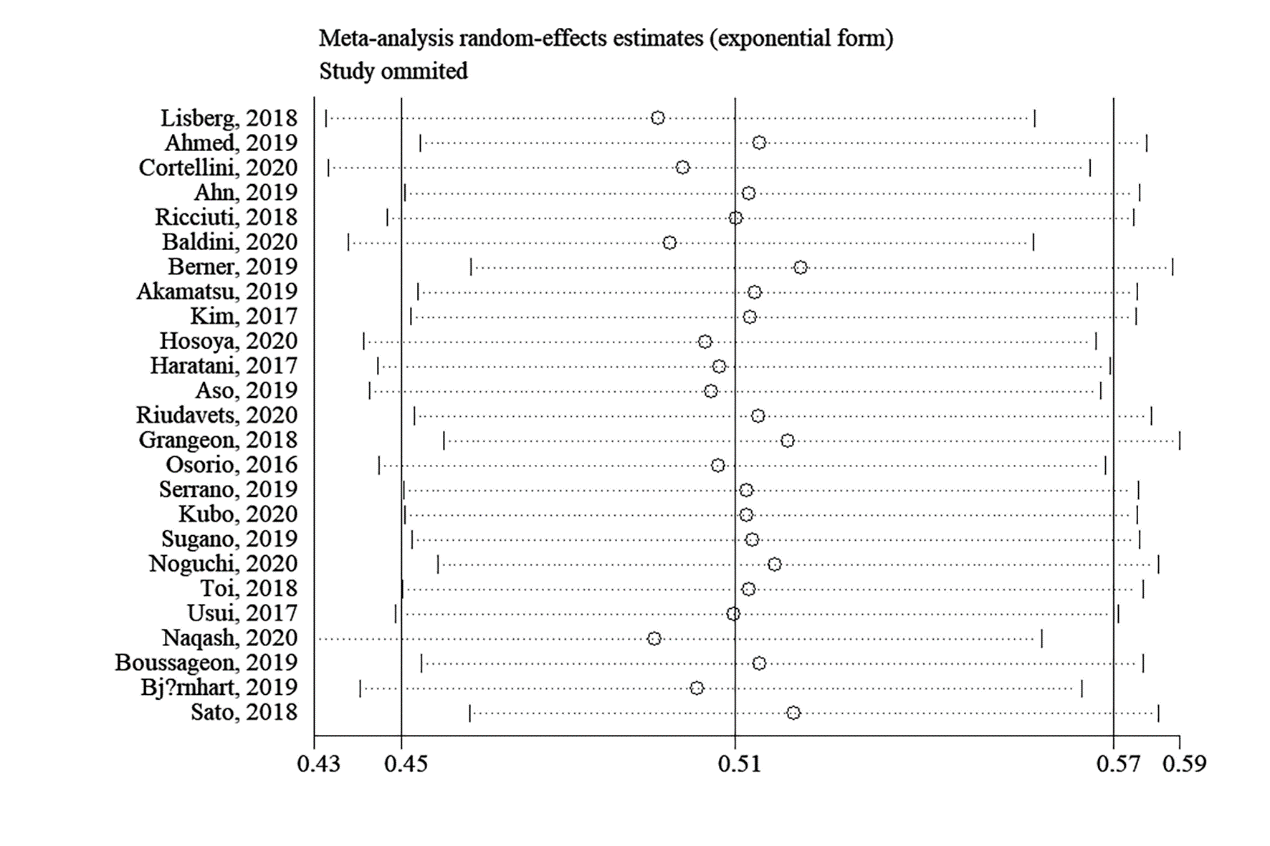
Figure S6: Sensitivity analysis of progression-free survival.


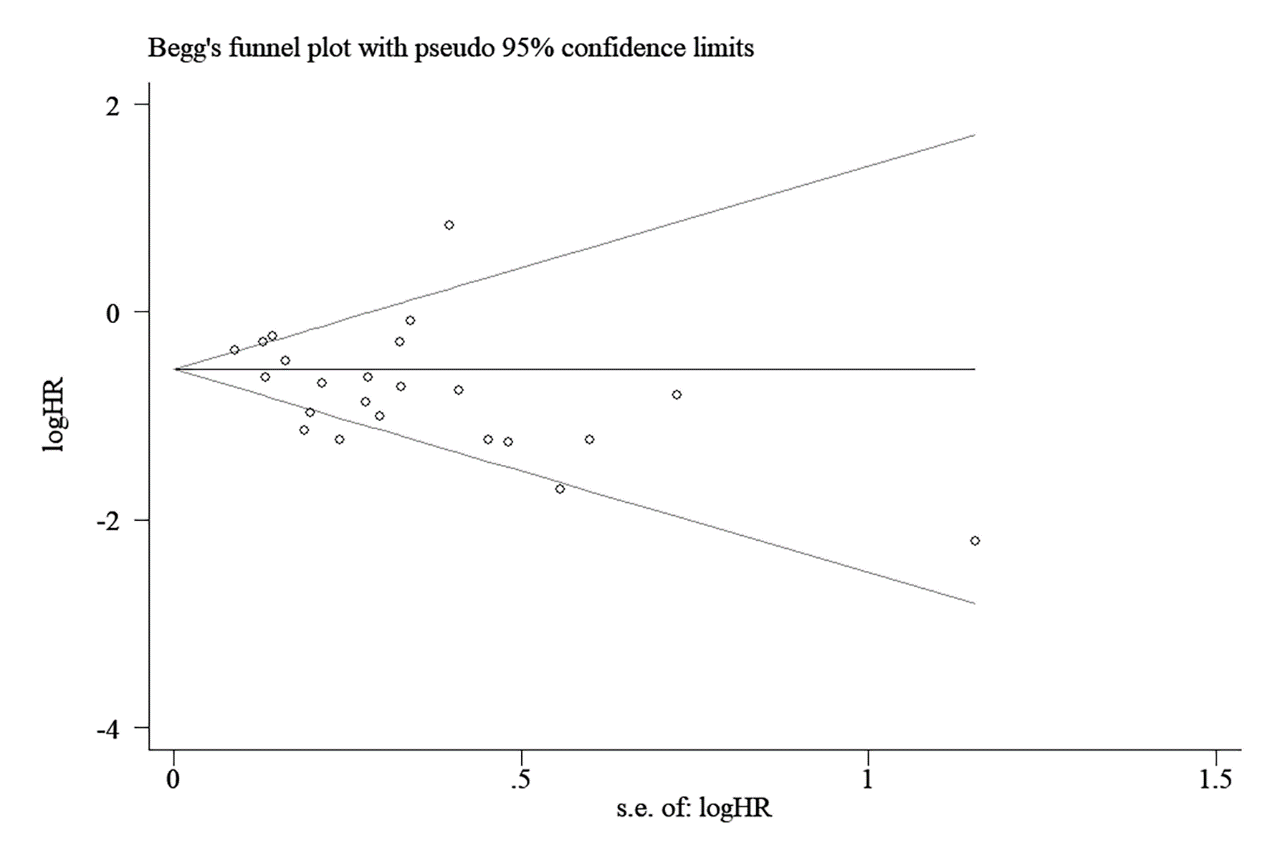


Figure S7: The funnel plots assessing the publication bias of overall survival. Abbreviation: HR, hazard ratio.


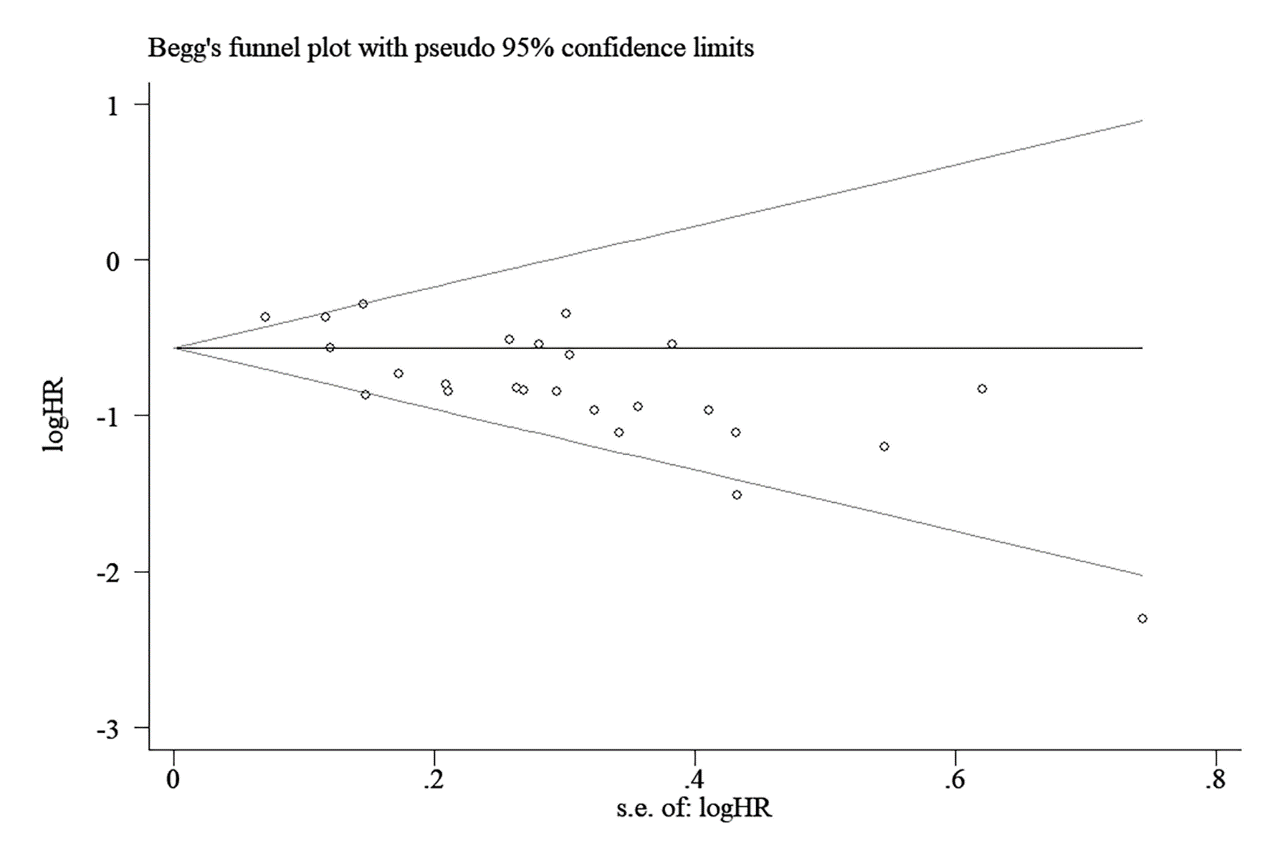


Figure S8: The funnel plots assessing the publication bias of progression-free survival. Abbreviation: HR, hazard ratio.
